# Trends and socioeconomic factors in smoking and alcohol consumption among Chinese people: evidence from the 2008–2018 National Health Service Surveys in Jiangsu Province

**DOI:** 10.1186/s13690-021-00646-9

**Published:** 2021-07-09

**Authors:** Kehui Liu, Yan Ding, Xiang Lu, Zhonghua Wang

**Affiliations:** 1 School of Health Policy Management, Nanjing Medical University-Nanjing, Nanjing, China; 2 Creative Health Policy Research Group, Nanjing Medical University, Nanjing, China; 3 Center for Global Health, Nanjing Medical University, Nanjing, China

**Keywords:** Smoking, Alcohol drinking, Socioeconomic factors, Public health

## Abstract

**Background:**

Smoking and excessive drinking are risk factors for many diseases. With the rapid economic development in China, it is important to identify trends in smoking and alcohol consumption and socioeconomic factors that contribute to these behaviors to ensure the health of the population.

**Methods:**

we analyzed pooled cross-sectional data from the fourth, fifth, and sixth National Health Service Surveys conducted in Jiangsu Province in 2008, 2013, and 2018, respectively. The study population was those over 15 years old in three surveys. Trends in smoking and alcohol use were analyzed with descriptive statistics, and bivariate and multinomial logistic regression was used to identify contributing factors.

**Results:**

Among total sample, smoking rate was 23.95%, in which the incidence of light, moderate and heavy smoking was 5.75, 4.63 and 13.56%, respectively; drinking rate was 23.29%, in which non-excessive drinking and excessive drinking were 19.80 and 3.49%, respectively, “smoking and drinking” rate was 13.41%. From 2008 to 2018, overall and light-to-moderate smoking rates first increased and then decreased while heavy smoking rate declined; alcohol consumption increased while excessive drinking increased before decreasing; and the incidence of “smoking and drinking” has been rising continuously. The trend of smoking and drinking rates in urban area was similar to rural area, however there was significant difference between urban and rural area. Socioeconomic factors, demographic, health-related and year variables were significant influencing factors of smoking and drinking.

**Conclusion:**

Our research can provide important evidences for tobacco and alcohol control in China and other similar developing countries. Preventive measures such as education and support services along with stricter regulations for tobacco and alcohol use are needed to improve public health in China.

## Background

Smoking and excessive alcohol consumption have adverse effects on individual and public health as well as negative economic and social consequences. Smoking is a risk factor for several diseases and a major cause of death [1]. Tobacco control measures recommended by the World Health Organization (WHO) cover about 5 billion people; however, 59 countries including China still fail to reach the highest level of implementation [2]. Meanwhile, alcohol use has been linked to diseases such as liver and esophageal cancers and cirrhosis, epilepsy, homicides, and motor vehicle accidents [3]. According to the WHO’s 2018 Global Status Report on Alcohol and Health, 3 million people worldwide—mostly men—die from alcohol-related diseases each year, constituting an enormous global public health burden. China’s per capita alcohol consumption increased by 80% between 2005 and 2018 [4].

Most countries recognize the importance of addressing the global tobacco and alcohol epidemic to protect the health of their citizens. In February 2005, the WHO promulgated the Framework Convention on Tobacco Control [5], and China issued the Health China 2030 plan in 2016 that outlined specific measures to promote healthy individual behaviors and lifestyles such as quitting smoking and limiting alcohol consumption [6]. According to the WHO, by the end of 2018, 125 countries or regions had formulated tobacco control laws or comprehensive legislation on smoking in public places [7]. As of August 2019, 22 cities in China had implemented local or national regulations to control smoking that cover 15% of the population [8]. The WHO’s 2010 Global Strategy to Reduce the Harmful Use of Alcohol represents an international consensus that reducing the harmful use of alcohol and its associated health and social burdens is a public health priority [9]. According to the WHO’s 2018 Global Survey on Alcohol and Health, 11 countries have banned the purchase or consumption of alcohol [4].

A prerequisite for the successful implementation of measures to prevent smoking and alcohol use is identifying factors that influence these behaviors [10]. Many studies have investigated the impact of socioeconomic factors on smoking and alcohol consumption, although they focused on different factors. For example, one study that analyzed data from 54 low- and middle-income countries found differences in tobacco use according to socioeconomic status in low-income countries and there was significant variability among countries, especially those with the lowest incomes [11]. It has been demonstrated that high education level has a positive effect on health and healthy behaviors, whereas a low level of education is positively correlated with unhealthy behaviors such as smoking and drinking [12, 13]. Socioeconomic factors such as occupation, education, and wealth were shown to be related to smoking, which was more common among individuals with a low-skill occupation and low education and income levels [14]. On the other hand, people in better health, with higher income and education levels, and who were more socially active were found to be more likely to consume harmful levels of alcohol [15]. There is also evidence that smoking and drinking behaviors are influenced by demographic factors such as age, sex, marital status, and location of residence; for example, studies conducted in China reported that people living in rural areas were more likely to smoke and drink than urban dwellers [16–18].

Most previous studies on the factors influencing smoking and drinking behavior have analyzed cross-sectional data, which do not reflect long-term trends [19–22]. Moreover, there is a large gap in development between urban and rural areas in China, but there have been few comparative analyses of smoking and drinking rates according to location and time trend. To address these issues, the present study analyzed trends in smoking and alcohol consumption rates and quantities in urban and rural areas as well as socioeconomic factors contributing to these behaviors based on National Health Service Survey (NHSS) data from Jiangsu Province, China. Therefore, it is of great significance to understand the trends and socioeconomic factors of smoking and drinking among residents in Jiangsu Province to provide evidence for tobacco control and alcohol restriction in China and other similar developing countries.

## Methods

### Data source

The primary data used in this study were derived from the Fourth (2008), Fifth (2013) and Sixth (2018) NHSS in Jiangsu Province. Since 1993, the NHSS has been organized every 5 years by the National Health Commission of China, with the provincial health commission being responsible for the survey of each region. The NHSS mainly consists of a household survey supplemented with an institutional survey; the former collects data through household interviews, with all permanent residents of surveyed households interviewed by trained and qualified investigators according to questionnaire items. A multistage stratified cluster random sampling method was used to select 156 counties (cities and districts) from 31 provinces in China; 5 towns (streets) were randomly selected in each of the sample counties (city or district); 2 villages (neighborhood committees) were randomly selected in each sample town (street), and 60 households were randomly selected in each sample village (neighborhood committees), for a total of 93,600 households (population of nearly 300,000).

Jiangsu Province is located in the Yangtze River economic belt, with a population of 80.7 million, 70.6 and 29.4% of the residents lived in urban and rural areas, respectively, which has the largest population density in China. At the end of 2019, both the per capita Gross Regional Product (GRP) and the regional Development and people’s Livelihood Index (DLI) of Jiangsu Province ranked first in China, becoming one of the provinces with the highest comprehensive development level in China.

Our research group was responsible for completing the National Health Service Survey of Jiangsu Province in 2008, 2013 and 2018, including 19 countries. In Jiangsu Province, the fourth NHSS was in June 2008, with 7021 respondents participating in the survey; the fifth NHSS was in June 2013, with 10,422 respondents; and the sixth NHSS was in September 2018, with 11,550 respondents. We used data from 2008, 2013, and 2018 to analyze trends in and socioeconomic factors contributing to smoking and alcohol consumption among Chinese people. The inclusion criteria were men and women over 15 years of age. After merging the NHSS datasets, the study population comprised 24,939 respondents. To ensure more accurate data analysis, respondents with any missing variables were excluded. NHSS data provides detailed information on demographic characteristics, socioeconomic status, health status, healthcare use and cost, health behaviors, etc. Strict quality control was implemented in every link of the national health service survey, including the quality control in the design stage (including the design of the questionnaire), the quality control of the investigators, the quality control in the field survey and in the data sorting, so as to fully ensure the quality of the survey [23].

### Variables selection

#### Dependent variable

The dependent variables were smoking, smoking quantity, drinking, drinking quantity and “smoking and drinking”. Smoking variable is a binary variable about whether smoking or not, which is determined by asking the respondents about their current smoking status. Respondents were also asked about their years of smoking and the average number of cigarettes smoked per day if they smoked. The smoking index (SI) was calculated as number of years of smoking × average number of cigarettes per day; smoking quantity was categorized as low/light smoking (SI ≤200), moderate (200 < SI < 400), or high/heavy smoking (SI ≥400) [21]. Drinking variable was a binary variable determined by asking the respondents whether they had drinking behavior in the past 6 months. Drinking quantity was converted to standard drinking units by the investigators. Specifically, 1 can of beer was equivalent to 1 drinking unit; 50 g of a beverage with < 40% alcohol was taken as equivalent to 1.5 drinking units; 50 g of a beverage with ≥40% alcohol was equivalent to 2 drinking units; 1 bottle of beer was equivalent to 2 drinking units; 500 g of wine was equivalent to 5 drinking units; and 500 g of yellow rice wine was equivalent to 6.5 drinking units. Drinking quantity was categorized as non-excessive or excessive drinking; the latter was defined as ≥5 and ≥ 4 drinking units at a time for men and women, respectively [24]. In addition, “smoking and drinking”, can be defined according to whether the reporter both smoking and drinking.

#### Independent variables

The independent variables were demographic information, socioeconomic status, and health-related information. Demographic variables (eg, age, sex, number of siblings, marital status, place of residence, and social health insurance) were included in our analyses in order to reduce the impact of differences between rural and urban populations. Socioeconomic status included income level, education level, employment status, type of occupation, and poor or low-security households. We used per capita annual income adjusted by price index as the income variable. Health-related variables included chronic disease, European Quality of Life Scale – 5 Dimensions (EQ-5D) score, health status, physical examination, and physical exercise. Self-reported health status was evaluated in the questionnaire with the Visual Analog Scale (VAS) and classified into 5 grades as in previous studies [25, 26]. Additionally, the wave variable (2008, 2013, and 2018) was included in order to account for fluctuations in smoking and drinking rates within each year of the NHSS. Detailed descriptions of dependent and independent variables are shown in Table 1.

**Table 1:** Description of explanatory variables

	Description	Indicator/survey questions
**Dependent variable**		
Smoking	0 = No	Question: What is your current smoking status?
	1 = Yes	
Smoking quantity	0 = No smoking	According to the smoking index (smoking index = number of cigarettes per day * number of years of smoking), the smoking amount is divided into light, moderate and heavy.
	1 = Low (light smoking)	
	2 = Moderate	
	3 = High (heavy smoking)	
Drinking	0 = No	Question: Did you drink in the past six months?
	1 = Yes	
Drinking volume	0 = No drinking	Judging whether to drink too much according to the 2007 standard of China chronic disease and its risk factors monitoring report.
	1 = Non-excessive drinking	
	2 = Excessive drinking	
Smoking and drinking	0 = No smoking or no drinking 1 = Both smoking and drinking	We extended a new dependent variable, both smoking and drinking, according to whether to smoke and whether to drink.
**Independent variable**		
Demographic factor		
Age, years	1 = 15–45	Question: Year of birth.
	2 = 46–59	
	3 = ≥60	
Sex	0 = Male	Question: What is your gender?
	1 = Female	
Number of siblings	1 = ≤2	Question: What is your household registration number?
	2 = 3 or 4	
	3 = ≥5	
Marital status	1 = Unmarried	Question: What is your present marital status?
	2 = Married	
	3 = Divorced/widowed/other	
Place of residence	0 = Urban	Question: Where do you live? Urban or rural areas?
	1 = Rural	
Social health insurance	0 = No	Question: Do you participate in social medical insurance?
	1 = Yes	
Socioeconomic status		
Income level	1 = Very low(≤12,453)	Yearly household income divided by the number of household members; first household member with a weight of 1, all following household members with a weight of 0.5.
	2 = Low(12,453 ~ 20,000)	
	3 = Middle(20,000 ~ 29,284)	
	4 = High(29,284 ~ 43,773)	
	5 = Very high(≥43,773)	
Education	1 = Primary school	Question: What’s the highest level of education in last waves?
	2 = Junior school	
	3 = High school	
	4 = University or higher	
Employment status	1 = Employed	Question: What is your present employment situation?
	2 = Retired	
	3 = Unemployed	
Type of occupation	1 = Unskilled labor	Question:What is your occupation type?
	2 = Skilled labor	
	3 = Other or unemployed	
Poor or low-security household	0 = No	Question: Is your family listed as a local poor or low-income family?
	1 = Yes	
Health-related factor		
Chronic disease	0 = No	Question: Have you had a chronic disease diagnosed by your doctor?
	1 = Yes	
Multiple chronic diseases	0 = No	“Yes” is defined as having two or more chronic diseases, and “no” is defined as not suffering from chronic diseases.
	1 = Yes	
EQ-5D	0 = Complete health	“Complete health” is defined as EQ-5D score equal to one point, while “incomplete health” is defined as EQ-5D score less than one point.
	1 = Incomplete health	
Health status (VAS, self-reported)	1 = Poor (0–40)	Health status on a 20 cm vertical scale with end points of 0 and 100 was asked on the day of the interview.
	2 = Moderate (41–60)	
	3 = Good (41–80)	
	0 = Excellent (81–100)	
Physical examination	0 = No	Question: have you had a physical examination in the past 12 months? (excluding examination due to illness)
	1 = Yes	
Physical exercise (no. times per week)	1 = 0	Question: on average, how many times a week do you exercise in the past 1 month?
	2 = 1 or 2	
	3 = 3–5	
	4 = ≥6	
Wave	1 = 2008	
	2 = 2013	
	3 = 2018	

*Abbreviations*: *EQ-5D* European Quality of Life Scale – 5 Dimensions, *VAS* Visual Analog Scale

### Data analysis

All statistical analyses were performed with Stata v14.0 software (Stata Corp, College Station, TX, USA) including descriptive statistics and bivariate and multinomial logistic regression analyses. *P* < 0.05 was statistically significant in all tests. We first evaluated whether there were statistically significant differences between respondents from urban and rural areas with the chi-squared test. We then calculated the rate and quantity of smoking and drinking in each year, and analyze the trends from 2008 to 2018. As smoking, drinking and “smoking and drinking” were binary variables, we used a bivariate logistic regression model to analyze factors influencing these behaviors based on the odds ratio (OR); and as smoking quantity and drinking quantity were multi-category variables, we used a multinomial logistic regression model to analyze the influencing factors based on the relative risk ratio (RRR).

## Results

### Descriptive statistical results

Table 2 presents statistics of dependent and independent variables by type of place of residence. Of the 24,939 respondents, 10,684 (42.84%) lived in urban areas and 14,255 (57.1%) in rural areas. The overall rate of smoking was 23.95%, with 5.75% light, 4.63% moderate, and 13.56% heavy smokers. Among residents of rural areas, 24.29% were smokers, with 5.55% light, 4.48% moderate, and 14.27% heavy smokers. In urban areas, 23.48% of the population smoked, with 6.03% light, 4.84% moderate, and 12.62% heavy smokers. Light and moderate smoking rates were significantly higher whereas the heavy smoking rate was lower in urban areas as compared to rural areas (*p* < 0.001). The overall rate of alcohol use was 23.29%, with 3.49% of the population engaging in excessive drinking. Drinking rates in urban and rural areas were 23.70 and 77.02%, respectively. The non-excessive drinking rate was higher in urban areas than in rural areas (20.83% vs 19.02%, *p* < 0.001), while the opposite was true for excessive drinking rate (2.86% vs 3.96%, *p* < 0.001). The overall incidence of “smoking and drinking” was 13.41%, and that of urban and rural areas was 13.58 and 13.28%, respectively.

**Table 2:** Characteristics of the study population in Jiangsu Province from 2008 to 2018

Variable	Total ***N*** = 24,939	Urban ***n*** = 10,684 (42.84%)	Rural ***n*** = 14,255 (57.10%)	***P*** value
Smoking				
No	18,967 (76.05%)	8175 (76.52%)	10,792 (75.71%)	
Yes	5972 (23.95%)	2509 (23.48%)	3463 (24.29%)	
Smoking quantity				***
Low (light smoking)	1435 (5.75%)	644 (6.03%)	791 (5.55%)	
Moderate	1155 (4.63%)	517 (4.84%)	638 (4.48%)	
High (heavy smoking)	3382 (13.56%)	1348 (12.62%)	2034 (14.27%)	
Drinking				
No	19,131 (76.71%)	8152 (76.30%)	10,979 (77.02%)	
Yes	5808 (23.29%)	2532 (23.70%)	3276 (22.98%)	
Drinking quantity				***
Non-excessive drinking	4932 (19.80%)	2226 (20.83%)	2712 (19.02%)	
Excessive drinking	873 (3.49%)	306 (2.86%)	564 (3.96%)	
Smoking and drinking				
No	21,595 (86.59%)	9233 (86.42%)	12,362 (86.72%)	
Yes	3344 (13.41%)	1451 (13.58%)	1893 (13.28%)	
**Demographic factor**				
Age, years				***
15–45	9954 (39.91%)	3905 (36.55%)	6049 (42.43%)	
46–59	7252 (29.08%)	2900 (27.14%)	4352 (30.53%)	
≥ 60	7733 (31.01%)	3879 (36.31%)	3854 (27.04%)	
Sex				
Male	12,200 (48.92%)	5211 (48.77%)	6989 (49.03%)	
Female	12,739 (51.08%)	5473 (51.23%)	7266 (50.97%)	
Number of siblings				***
≤ 2	6907 (27.70%)	3470 (32.48%)	3437 (24.11%)	
3 or 4	10,738 (43.06%)	4705 (44.04%)	6033 (42.32%)	
≥ 5	7294 (29.25%)	2509 (23.48%)	4785 (33.57%)	
Marital status				*
Unmarried	2520 (10.10%)	1089 (10.19%)	1431 (10.04%)	
Married	20,516 (82.26%)	8727 (81.68%)	11,789 (82.70%)	
Divorced/widowed/other	1903 (7.63%)	868 (8.12%)	1035 (7.26%)	
Social health insurance				
No	862 (3.46%)	394 (3.69%)	468 (3.28%)	
Yes	24,077 (96.54%)	10,290 (96.31%)	13,787 (96.72%)	
**Socioeconomic status**				
Income level				***
Very low	5522 (22.14%)	657 (6.15%)	4865 (34.13%)	
Low	4518 (18.12%)	1291 (12.08%)	3227 (22.64%)	
Middle	4921 (19.73%)	2233 (20.90%)	2688 (18.86%)	
High	5535 (22.19%)	3291 (30.80%)	2244 (15.74%)	
Very high	4443 (17.82%)	3212 (30.06%)	1231 (8.64%)	
Education				***
Primary school	8327 (33.39%)	2256 (21.12%)	6071 (42.59%)	
Junior school	8564 (34.34%)	3393 (31.76%)	5171 (36.27%)	
High school	4711 (18.89%)	2557 (23.93%)	2154 (15.11%)	
University or higher	3337 (13.38%)	2478 (23.19%)	859 (6.03%)	
Employment status				***
Employed	16,521 (66.25%)	5369 (50.25%)	11,152 (78.23%)	
Retired	3926 (15.74%)	3474 (32.52%)	452 (3.17%)	
Unemployed	4492 (18.01%)	1841 (17.23%)	2651 (18.60%)	
Type of occupation				***
Unskilled labor	5236 (21.00%)	3979 (37.24%)	1257 (8.82%)	
Skilled labor	14,078 (56.45%)	4154 (38.88%)	9924 (69.62%)	
Other or unemployed	5625 (22.56%)	2551 (23.88%)	3074 (21.56%)	
Poor or low-security household^†^				***
No	24,036 (96.38%)	10,407 (97.41%)	13,629 (95.61%)	
Yes	903 (3.62%)	277 (2.59%)	626 (4.39%)	
**Health related factor**				
Chronic disease				***
No	17,048 (68.36%)	6706 (62.77%)	10,342 (72.55%)	
Yes	7891 (31.64%)	3978 (37.23%)	3913 (27.45%)	
Multiple chronic diseases				***
No	23,067 (92.49%)	9664 (90.45%)	13,403 (94.02%)	
Yes	1872 (7.51%)	1020 (9.55%)	852 (5.98%)	
EQ-5D				
Complete health	20,721 (83.09%)	8829 (82.64%)	11,892 (83.42%)	
Incomplete health	4218 (16.91%)	1855 (17.36%)	2363 (16.58%)	
Health status				***
Poor (0–40)	345 (1.39%)	136 (1.27%)	209 (1.46%)	
Moderate (41–60)	2388 (9.58%)	1052 (9.85%)	1336 (9.37%)	
Good (41–80)	9910 (39.74%)	4542 (42.51%)	5368 (37.66%)	
Excellent (81–100)	12,296 (49.30%)	4954 (46.37%)	7342 (51.50%)	
Physical examination				***
No	13,297 (53.32%)	4557 (42.65%)	8740 (61.31%)	
Yes	11,642 (46.68%)	6127 (57.35%)	5515 (38.69%)	
Physical exercise (no. times per week)				***
0	15,898 (63.75%)	4792 (44.85%)	11,106 (77.91%)	
1 or 2	1858 (7.45%)	1104 (10.33%)	754 (5.29%)	
3–5	2304 (9.24%)	1346 (12.60%)	958 (6.72%)	
≥ 6	4879 (19.56%)	3442 (32.22%)	1437 (10.08%)	
**Wave**				***
2008	5987 (24.01%)	1385 (12.96%)	4602 (32.28%)	
2013	9194 (36.87%)	4208 (39.39%)	4986 (34.98%)	
2018	9758 (39.13%)	5091 (47.65%)	4667 (32.74%)	

*P < 0.05, **P < 0.01, ***P < 0.001

^†^Poor or low-security households are low-income groups subsidized by the government

*Abbreviation*: *EQ-5D* European Quality of Life Scale – 5 Dimensions

Rural and urban populations differed significantly with respect to most of the independent variables. Education level and personal annual income were significantly higher for urban respondents than for rural respondents: the proportions of respondents with high or very high income were 30.8 and 30.06%, respectively, for the former group and 15.74 and 8.64%, respectively, for the latter. The proportions of urban respondents with a high school education or university or higher education level were 23.93 and 23.19%, respectively, which were significantly higher than the proportions of rural respondents (15.11 and 6.03%, respectively). The rates of employment and unemployment were significantly lower in urban areas (50.25 and 17.23%, respectively) than in rural areas (78.23 and 18.6%, respectively), while the proportion of retirees was higher in urban as compared to rural areas (32.52% vs 3.17%). The proportion of poor or low-security households was larger in rural areas (4.39%) than in urban areas (2.59%). In terms of health-related variables, the rate of chronic diseases was higher in the urban population (37.23%) than among rural residents (27.45%); the latter were also less likely to have participated in physical examinations (38.69% vs 57.35%).

### Trends in smoking and alcohol consumption

**Fig. 1:**
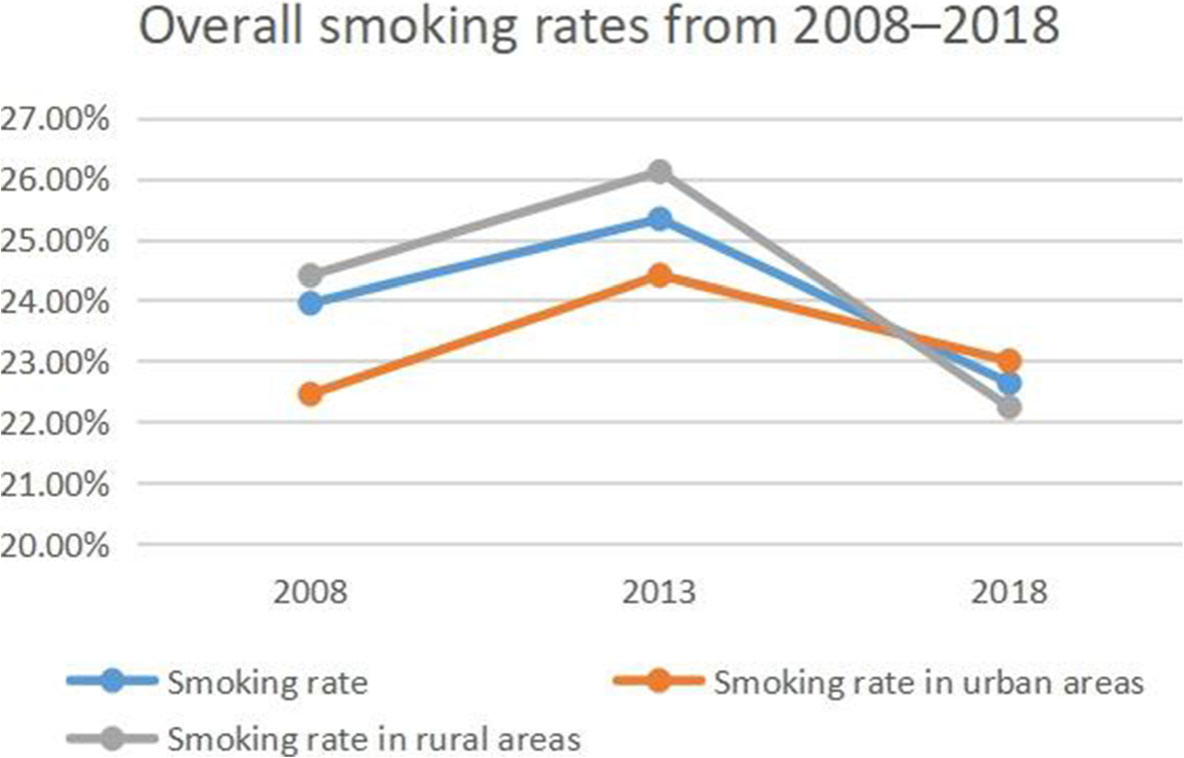
Overall smoking rates from 2008 to 2018 in Jiangsu province

**Fig. 2:**
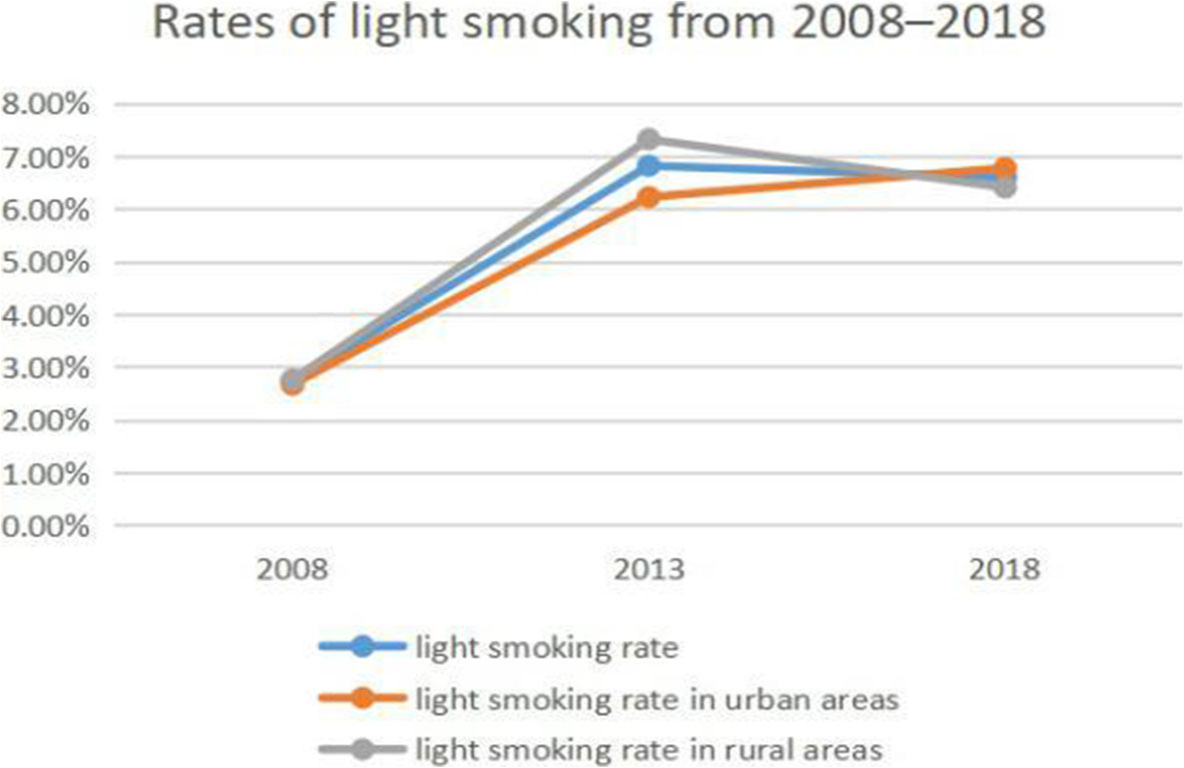
Rates of light smoking from 2008 to 2018 in Jiangsu province

**Fig. 3:**
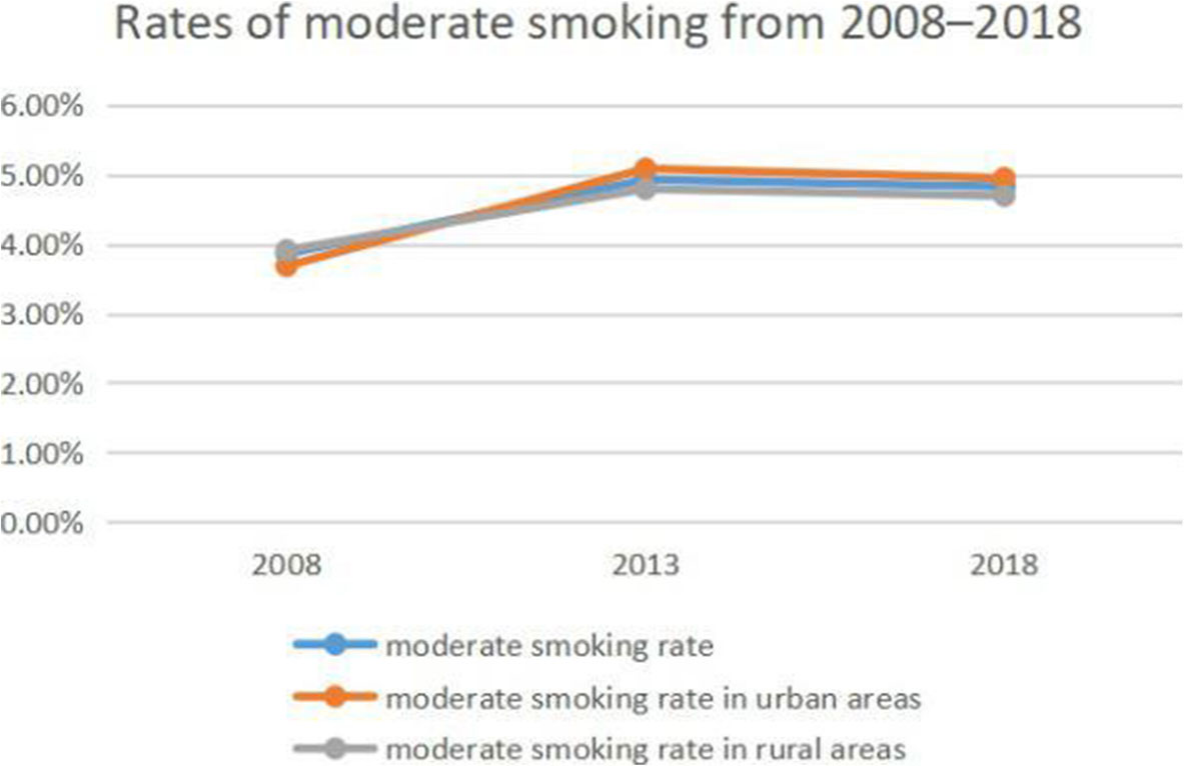
Rates of moderate smoking from 2008 to 2018 in Jiangsu province

**Fig. 4:**
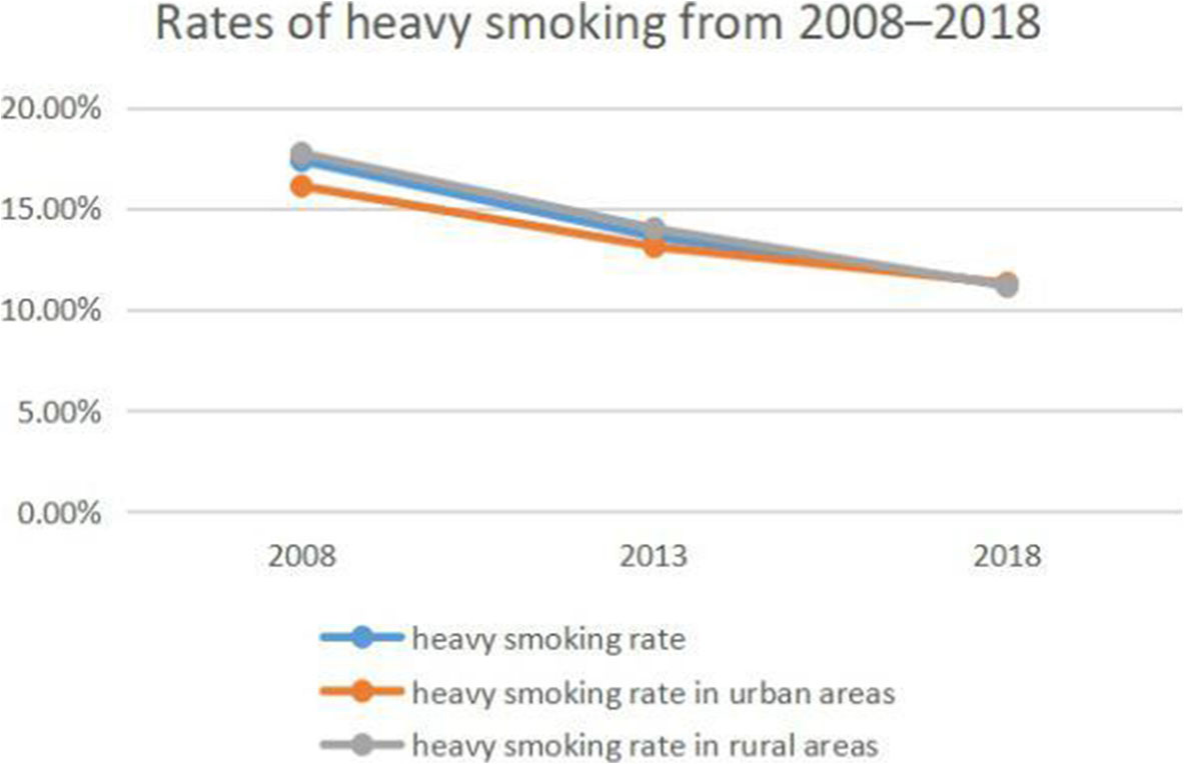
Rates of heavy smoking from 2008 to 2018 in Jiangsu province

The smoking rate increased from 23.95% in 2008 to 25.33% in 2013 but decreased to 25.33% in 2018 (Fig. 1). In 2008 and 2013, the smoking rate was higher in rural areas (26.11 and 24.4%, respectively) than in urban areas (24.41 and 22.45%, respectively), but this was reversed in 2018 (22.24% vs 23%). Similar trends were observed in light and moderate smoking rates (Figs. 2 and 3). However, the rate of heavy smoking showed a continuously declining trend over time (Fig. 4). Additionally, while the rates of light, moderate, and heavy smoking were higher in rural areas as compared to urban areas in 2008 (light: 2.76% vs 2.67%; moderate: 3.91% vs 3.68%; heavy: 17.73% vs 16.1%), the reverse was true in 2018 (light: 6.41% vs 6.78%; moderate: 4.69% vs 4.95%; heavy: 11.14% vs 11.27%).

**Fig. 5:**
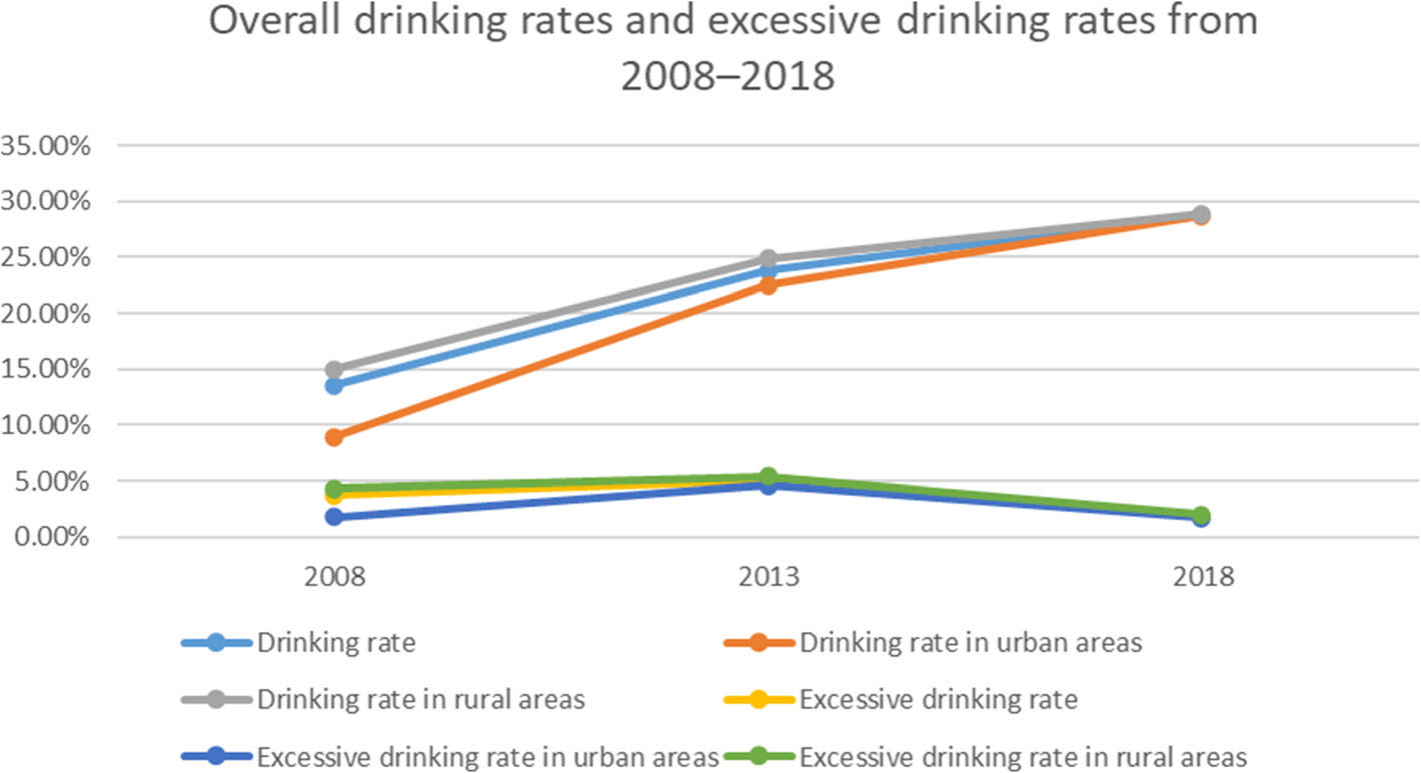
Overall drinking rates and excessive drinking rates from 2008 to 2018 in Jiangsu province

Drinking rates increased from 13.58% in 2008 to 23.8% in 2013 and 28.77% in 2018 (Fig. 5). The rate was higher in rural areas than in urban areas in 2008 (14.97% vs 8.95%) and 2013 (24.87% vs 22.53%), but was comparable between the 2 locations in 2018 (rural: 28.86% vs urban: 28.68%). The overall rate of excessive drinking increased from 3.76% in 2008 to 5.06% in 2013, and then decreased to 1.88% in 2018 (Fig. 5). Similar trends were observed in urban areas (2008: 1.81%, 2013: 4.61%; 2018: 1.71%) and rural areas (2008: 4.32%; 2013: 5.42%; 2018: 2.04%). It is worth noting that drinking and excessive drinking rates were significantly higher in rural as compared to urban areas throughout the survey period; however, the rates in urban areas increased more dramatically from 2008 to 2013, such that the differences between urban and rural areas shrank from 2013 to 2018.

**Fig. 6:**
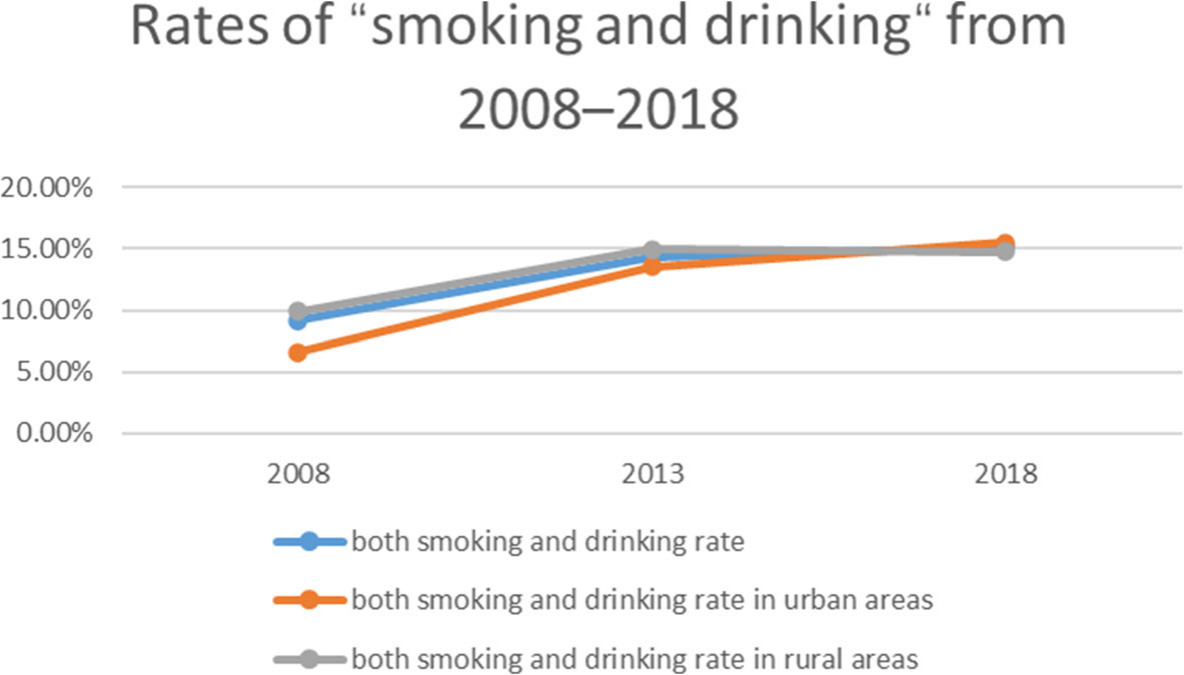
Rates of “smoking and drinking” from 2008 to 2018 in Jiangsu province

The incidence of “smoking and drinking” increased from 2008 to 2013, and then from 2013 to 2018, regardless of the overall incidence, urban incidence or rural incidence (Fig. 6). The overall incidence increased from 9.17% in 2008 to 14.32% in 2013, and then to 15.15% in 2018; the urban incidence increased from 6.64% in 2008 to 13.55% in 2013, and then to 15.50% in 2018; the rural incidence increased from 9.93% in 2008 to 14.98% in 2013, and then to 14.76% in 2018. The incidence of “smoking and drinking” in rural areas was higher than that in urban areas in 2008 and 2013, and that in urban areas was higher than that in rural areas in 2018.

### Influencing factors of smoking rate and quantity

After controlling for confounding variables, we found that smoking rate and quantity differed significantly between rural and urban areas (Table 3). Rural respondents were 30% less likely to smoke (OR = 0.7), 22% less likely to be light smokers (RRR = 0.78), 36% less likely to be moderate smokers (RRR = 0.64), and 30% less likely to be heavy smokers (RRR = 0.7). Other demographic factors also influenced smoking rate and quantity. Older respondents (≥46 years) were more likely to smoke, less likely to be light or moderate smokers, and more likely to be heavy smokers than respondents who were ≤ 45 years old. Smoking was less common in women than in men (OR = 0.02), and the rates of light, moderate, and heavy smoking were lower in women than in men (RRR = 0.18, 0.01, and 0.01, respectively). Married people were more likely to smoke than those who were unmarried (OR = 2.56); this was true for light (RRR = 1.24), moderate (RRR = 10.07), and heavy (RRR = 4.53) smoking. People with social health insurance were less likely to smoke (OR = 0.7), whether lightly (RRR = 0.58), moderately (RRR = 0.72), or heavily (RRR = 0.7). Socioeconomic status influenced smoking rate and quantity: compared to people with very low income, those with a very high income were more likely to smoke (OR = 1.18) and to be moderate (RRR = 1.26) or heavy (RRR = 1.28) smokers. There was a significant inverse correlation between smoking rate and education level, with an especially close correlation observed for the rate of heavy smoking. Compared to respondents who were employed, those who were unemployed or retired people were ~ 50% less likely to smoke, whether lightly, moderately, or heavily.

**Table 3:** Results of regression analysis of smoking rate and quantity in Jiangsu province from 2008 to 2018

Variable	Smoking		Light smoking		Moderate smoking		Heavy smoking	
	OR	95% CI	RRR	95% CI	RRR	95% CI	RRR	95% CI
**Demographic factor**								
Age, years (ref: 15–45)								
46–59	1.43***	(1.29,1.58)	0.34***	(0.28,0.40)	0.80***	(0.68,0.95)	4.92***	(4.27,5.68)
≥ 60	1.43***	(1.25,1.64)	0.25***	(0.19,0.33)	0.58***	(0.45,0.75)	5.40***	(4.52,6.45)
Sex (ref: male)								
Female	0.02***	(0.01,0.02)	0.18***	(0.01,0.02)	0.01***	(0.01,0.02)	0.01***	(0.01,0.02)
Number of siblings (ref: 1 or 2)								
3 or 4	0.99	(0.90,1.09)	0.84*	(0.70,1.01)	1.04	(0.88,1.24)	1.05	(0.93,1.17)
≥ 5	1.02	(0.91,1.11)	1.07	(0.90,1.30)	0.86	(0.71,1.04)	0.92	(0.81,1.03)
Marital status (ref: unmarried)								
Married	2.56***	(2.21,2.97)	1.24**	(1.03,1.50)	10.07***	(6.01,15.58)	4.53***	(3.41,6.03)
Divorced/widowed/other	3.27***	(2.64,4.04)	1.77***	(1.24,2.53)	12.41***	(7.40,20.84)	5.13***	(3.69,7.13)
Place of residence (ref: urban)								
Rural	0.70***	(0.64,0.77)	0.78***	(0.67,0.90)	0.64***	(0.54,0.75)	0.70***	(0.62,0.79)
Social health insurance (ref: no)								
Yes	0.70***	(0.57,0.86)	0.58***	(0.43,0.77)	0.72*	(0.51,1.02)	0.70**	(0.53,0.92)
**Socioeconomic status**								
Income (ref: very low)								
Low	1.02	(0.90,1.15)	1.03	(0.84,1.28)	0.99	(0.79,1.23)	1.02	(0.88,1.18)
Middle	1.02	(0.90,1.16)	1.04	(0.83,1.29)	0.89	(0.71,1.13)	1.03	(0.88,1.20)
High	1.06	(0.93,1.21)	0.96	(0.77,1.20)	1.06	(0.84,1.33)	1.1	(0.94,1.29)
Very high	1.18**	(1.02,1.40)	1	(0.78,1.27)	1.26*	(0.98,1.62)	1.28***	(1.07,1.53)
Education (ref: primary school)								
Junior school	0.85***	(0.77,0.94)	1.03	(0.84,1.26)	1.19*	(0.99,1.44)	0.83***	(0.74,0.93)
High school	0.68***	(0.60,0.77)	1.11	(0.89,1.39)	0.80**	(0.63,1.00)	0.56***	(0.48,0.65)
University or higher	0.30***	(0.25,0.36)	0.55***	(0.42,0.72)	0.24***	(0.17,0.33)	0.18***	(0.14,0.23)
Employment status (ref: employed)								
Retired	0.49***	(0.43,0.57)	0.52***	(0.38,0.72)	0.48***	(0.36,0.64)	0.54***	(0.46,0.64)
Unemployed	0.51***	(0.42,0.63)	0.33***	(0.23,0.48)	0.57***	(0.39,0.83)	0.60***	(0.46,0.77)
Type of occupation (ref: unskilled labor)								
Skilled labor	0.83***	(0.75,0.92)	0.87*	(0.74,1.02)	0.80**	(0.67,0.96)	0.87*	(0.76,1.00)
Unemployed or other	1.03	(0.85,1.24)	0.82	(0.61,1.10)	1.04	(0.76,1.46)	1.16	(0.93,1.46)
Poor or low-security household (ref: no)								
Yes	1.05	(0.86,1.29)	0.89	(0.61,1.30)	0.83	(0.55,1.25)	1.21+	(0.96,1.53)
**Health-related factor**								
Chronic disease (ref: no)								
Yes	0.94	(0.86,1.03)	0.93	(0.77,1.11)	1.02	(0.86,1.21)	0.95	(0.85,1.06)
Multiple chronic diseases (ref: no)								
Yes	0.78***	(0.67,0.92)	0.99	(0.71,1.38)	0.83	(0.61,1.11)	0.75***	(0.63,0.90)
EQ-5D (ref: complete health)								
Incomplete health	0.81***	(0.72,0.91)	0.98	(0.78,1.23)	0.94	(0.76,1.16)	0.75***	(0.65,0.85)
Health status (ref: excellent)								
Poor (0–40)	0.57***	(0.39,0.82)	0.81	(0.39,1.70)	0.56	(0.27,1.16)	0.49***	(0.32,0.77)
Moderate (41–60)	0.95	(0.81,1.09)	1	(0.74,1.35)	0.96	(0.73,1.27)	0.93	(0.78,1.11)
Good (41–80)	1.08	(0.99,1.17)	1.01	(0.88,1.16)	0.9	(0.78,1.05)	1.15***	(1.03,1.27)
Physical examination (ref: no)								
Yes	0.91**	(0.84,0.98)	1	(0.88,1.13)	0.92	(0.80,1.06)	0.90**	(0.81,0.99)
Physical exercise, no. of times per week (ref: ≥6)								
0	1.35***	(1.21,1.50)	1.12	(0.94,1.34)	1.24**	(1.02,1.50)	1.47***	(1.29,1.68)
1 or 2	1.24**	(1.05,1.46)	1.05	(0.82,1.35)	1.31*	(0.99,1.73)	1.25**	(1.00,1.56)
3–5	0.94	(0.81,1.10)	0.87	(0.68,1.11)	0.97	(0.74,1.27)	0.94	(0.77,1.14)
**Wave** (ref: 2008)								
2013	1.09	(0.98,1.21)	3.29***	(2.67,4.04)	1.40***	(1.14,1.71)	0.62***	(0.55,0.71)
2018	0.98	(0.88,1.10)	3.10***	(2.52,3.83)	1.33***	(1.08,1.63)	0.52***	(0.46,0.60)

*P < 0.05, **P < 0.01, ***P < 0.001

*Abbreviations*: *CI* confidence interval, *EQ-5D* European Quality of Life Scale – 5 Dimensions, *OR* odds ratio, *ref.* reference, *RRR* relative risk ratio

People with multiple chronic diseases were less likely to smoke (OR = 0.78) and smoke heavily (RRR = 0.75). Compared to respondents who were in very good health, people with poor health were significantly less likely to smoke (OR = 0.57) and smoke heavily (RRR = 0.49). EQ-5D scores also indicated that the rates of smoking (OR = 0.81) and heavy smoking (RRR = 0.75) were significantly lower in respondents with incomplete health than in those with complete health. Compared to people who did not have regular physical examinations, those who had undergone a physical examination in the previous 12 months were less likely to smoke (OR = 0.91) and smoke heavily (RRR = 0.9). People who did not engage in physical exercise were more likely to smoke (OR = 1.35)—including smoking moderately (RRR = 1.24) and heavily (RRR = 1.47)—than those who exercised regularly.

### Influencing factors of drinking rate and quantity

Demographic variables including age, sex, and marital status were significant influencing factors of drinking rate and quantity (Table 4). People ≥46 years old were more likely to consume alcohol than those ≤45 years old, whereas the 46–59 year age group was more likely to drink excessively than people ≤45 years (RRR = 1.55). Women were less likely to drink than men (OR = 0.05), including drinking excessively (RRR = 0.01). Married people were more likely to drink than those who were unmarried people (OR = 3.26) and to engage in non-excessive (RRR = 3.2) and excessive (RRR = 3.49) drinking. Alcohol consumption was positively correlated with income level and negatively correlated with education level. People with high school-level education or higher were less likely to drink than those with primary school or lower education, whether this consisted of non-excessive or excessive drinking. Compared to employed respondents, those who were retired (OR = 0.64) or unemployed (OR = 0.73) were less likely to drink and to drink excessively (RRR = 0.54, RRR = 0.57).

**Table 4:** Results of regression analysis of drinking rate and quantity in Jiangsu province from 2008 to 2018

Variable	Drinking		Non-excessive drinking		Excessive drinking	
	OR	95% CI	RRR	95%CI	RRR	95%CI
**Demographic factor**						
Age, years (ref: 15–45)						
46–59	1.70***	(1.54,1.88)	1.73***	(1.56,1.93)	1.55***	(1.28,1.87)
≥ 60	1.41***	(1.23,1.61)	1.53***	(1.33,1.76)	0.87	(0.66,1.15)
Sex (ref: male)						
Female	0.05***	(0.04,0.05)	0.05***	(0.05,0.06)	0.01***	(0.01,0.02)
Number of siblings (ref: 1 or 2)						
3 or 4	0.96	(0.88,1.06)	0.98	(0.89,1.08)	0.86	(0.71,1.03)
≥ 5	0.94	(0.85,1.03)	0.95	(0.85,1.05)	0.86	(0.70,1.06)
Marital status (ref: unmarried)						
Married	3.26***	(2.78,3.82)	3.20***	(2.70,3.74)	3.49***	(2.45,4.98)
Divorced/widowed/other	2.81***	(2.26,3.49)	2.83***	(2.25,3.56)	2.39**	(1.43,3.99)
Place of residence (ref: urban)						
Rural	1.02	(0.94,1.12)	1	(0.92,1.10)	1.15	(0.95,1.38)
Social health insurance (ref: no)						
Yes	0.92	(0.75,1.14)	0.93	(0.74,1.17)	0.9	(0.60,1.35)
**Socioeconomic status**						
Income (ref: very low)						
Low	1.20***	(1.07,1.36)	1.18**	(1.04,1.34)	1.32**	(1.04,1.68)
Middle	1.31***	(1.16,1.49)	1.30***	(1.14,1.48)	1.42***	(1.11,1.82)
High	1.36***	(1.19,1.54)	1.33***	(1.16,1.52)	1.51***	(1.16,1.96)
Very high	1.39***	(1.21,1.60)	1.32***	(1.14,1.53)	1.94***	(1.45,2.60)
Education (ref: primary school)						
Junior school	0.94	(0.85,1.03)	0.96	(0.86,1.06)	0.82**	(0.67,1.00)
High school	0.75***	(0.67,0.85)	0.77***	(0.68,0.87)	0.67***	(0.53,0.86)
University or higher	0.58***	(0.50,0.69)	0.63***	(0.54,0.75)	0.31***	(0.26,0.53)
Employment status (ref: employed)						
Retired	0.64***	(0.56,0.74)	0.67***	(0.57,0.76)	0.54***	(0.39,0.75)
Unemployed	0.73***	(0.60,0.89)	0.77**	(0.63,0.96)	0.57***	(0.38,0.84)
Type of occupation (ref: unskilled labor)						
Skilled labor	0.93	(0.84,1.03)	0.95	(0.86,1.06)	0.81**	(0.66,0.99)
Unemployed or other	0.88	(0.74,1.06)	0.84*	(0.69,1.02)	1.02	(0.74,1.40)
Poor or low-security household (ref: no)						
Yes	0.88	(0.71,1.08)	0.85	(0.69,1.06)	1.03	(0.67,1.58)
**Health-related factor**						
Chronic disease (ref: no)						
Yes	1.01	(0.94,1.12)	0.99	(0.90,1.08)	1.18*	(0.98,1.41)
Multiple chronic diseases (ref: no)						
Yes	0.73***	(0.63,0.85)	0.76***	(0.65,0.90)	0.49***	(0.33,0.73)
EQ-5D (ref: complete health)						
Incomplete health	1.05	(0.94,1.18)	1.08	(0.96,1.21)	0.83	(0.63,1.09)
Health status (ref: excellent)						
Poor (0–40)	0.35***	(0.24,0.52)	0.39***	(0.26,0.58)	0.09**	(0.01,0.66)
Moderate (41–60)	0.63***	(0.54,0.74)	0.62***	(0.53,0.72)	0.78+	(0.56,1.09)
Good (41–80)	0.92**	(0.85,0.99)	0.93	(0.86,1.02)	0.85**	(0.72,1.00)
Physical examination (ref: no)						
Yes	1.05	(0.97,1.13)	1.05	(0.97,1.13)	1.06	(0.90,1.24)
Physical exercise, no. of times per week (ref: ≥6)						
0	1.10 *	(0.99,1.22)	1.08	(0.97,1.20)	1.25*	(0.99,1.57)
1 or 2	1.17*	(1.00,1.36)	1.19**	(1.02,1.40)	1	(0.69,1.45)
3–5	0.91	(0.78,1.05)	0.9	(0.77,1.04)	1	(0.72,1.39)
**Wave** (ref: 2008)						
2013	2.11***	(1.89,2.36)	2.29***	(2.03,2.59)	1.63***	(1.33,1.99)
2018	3.29***	(2.94,3.68)	4.13***	(3.66,4.67)	0.83	(0.65,1.05)

Chronic disease, health status, and physical exercise were also significant influencing factors of drinking rate and quantity. People with a chronic disease were significantly more likely to drink excessively than those without a chronic disease (RRR = 1.18), while those with ≥2 chronic diseases were significantly less likely to drink than those without chronic diseases (OR = 0.73), whether the drinking was excessive (RRR = 0.49) or not (RRR = 0.76). A lower level of health was associated with a lower alcohol consumption rate. People who did little physical exercise were more likely to drink and drink excessively than those who exercised regularly (OR = 1.10, RRR = 1.25).

### Influencing factors of smoking and drinking

Table 5 shows the logistic regression results of “both smoking and drinking”. Demographic variables including age, sex, marital status and place of residence were significant influencing factors of both smoking and drinking behavior. People aged 46–59(OR = 1.49) and over 60(OR = 1.17) were significantly more likely to both smoke and drink. Women were significantly less likely to smoke and drink than men (OR = 0.01). In terms of marital status, compared with unmarried people, married (OR = 3.07), divorced, widowed and other people (OR = 2.87) were significantly more likely to smoke and drink. Residence was also one of the significant factors influencing both smoking and drinking behavior, people in rural areas were significantly less likely to smoke and drink (OR = 0.82).

**Table 5:** Results of regression analysis of “both smoking and drinking” in Jiangsu province from 2008 to 2018

Variables	smoking and drinking	
	OR	95% CI
**Demographic factor**		
Age, years (ref: 15–45)		
46–59	1.49***	(1.33,1.67)
≥ 60	1.17*	(0.005, 0.01)
Sex (ref: male)		
female	0.01***	(0.01,0.02)
Number of siblings (ref: 1 or 2)		
3 or 4	1.03	(0.92,1.15)
≥ 5	1.06	(0.94,1.18)
Marital status (ref: unmarried)		
Married	3.07***	(2.51,3.76)
Divorced/widowed/other	2.87***	(2.19,3.77)
Place of residence (ref: urban)		
Rural	0.82***	(0.74,0.91)
Social health insurance (ref: no)		
Yes	0.81	(0.64,1.03)
**Socioeconomic status**		
Income (ref: very low)		
Low	1.09	(0.95,1.25)
Middle	1.14	(0.99,1.32)
High	1.11	(0.96,1.29)
Very high	1.28**	(1.08,1.51)
Education (ref: primary school)		
Junior school	0.89*	(0.79,0.99)
High school	0.70***	(0.61,0.80)
University or higher	0.39***	(0.32,0.47)
Employment status (ref: employed)		
Retired	0.64***	(0.54,0.76)
Unemployed	0.61***	(0.48,0.78)
Type of occupation (ref: unskilled labor)		
Skilled labor	0.89	(0.79,1.01)
Unemployed or other	1.01	(0.82,1.23)
Poor or low-security household (ref: no)		
Yes	1.12	(0.89,1.42)
**Health-related factor**		
Chronic disease (ref: no)		
Yes	0.96	(0.86,1.07)
Multiple chronic diseases (ref: no)		
Yes	0.74**	(0.62,0.90)
EQ-5D (ref: complete health)		
Incomplete health	0.88	(0.77,1.01)
Health status (ref: excellent)		
Poor (0–40)	0.34***	(0.20,0.58)
Moderate (41–60)	0.73**	(0.61,0.88)
Good (41–80)	0.98	(0.89,1.07)
physical examination (ref: no)		
Yes	0.94	(0.86,1.03)
Physical exercise, no. of times per week (ref: ≥6)		
0	1.25***	(1.11,1.41)
1 or 2	1.23**	(1.02,1.48)
3–5	0.83*	(0.70,0.99)
**Wave** (Ref: 2008)		
2013	1.78***	(1.56, 2.03)
2018	2.17***	(1.90, 2.47)

Socioeconomic status including income, education and employment status were significantly related to both smoking and drinking. People with very high income were 28%(*P <* 0.01) more likely to both smoke and drink than people with very low income. Education level was negatively associated with both smoking and drinking, people with junior school degree (OR = 0.89), high school degree (OR = 0.70) and university or higher degree (OR = 0.39) were all significantly less likely to smoke and drink than people with primary school education, and the odds ratio decreased with the improvement of educational level. Compared with the employed, the retired (OR = 0.64) and unemployed (OR = 0.61) were around 40%(*P <* 0.001) less likely to both smoke and drink.

Multiple chronic diseases, health status and physical exercise were also significant influencing factors of both smoking and drinking. People with multiple chronic diseases were significantly less likely to smoke and drink (OR = 0.74). Compared with the excellent health group, the moderate and poor health groups were significantly less likely to both smoke and drink, and the odds ratio of the poor health group (OR = 0.34) was lower than that of the moderate health group (OR = 0.73). Compared with people who exercise more than five times a week, people who exercise 3–5 times were less likely to both smoke and drink (OR = 0.83), while people who exercise less than two times are more likely to both smoke and drink(1 or 2 times: OR = 1.23; 0 time: OR = 1.25).

Moreover, people in 2013 and 2018 were significantly more likely to both smoke and drink in comparison with 2008, and the odds ratio was estimated to be higher in 2018.

## Discussion

In this study, we analyzed pooled cross-sectional data from the NHSS of Jiangsu Province to identify trends and socioeconomic factors in smoking and alcohol consumption among Chinese people. The overall smoking rate and light and moderate smoking rates increased from 2008 to 2013 and then decreased from 2013 to 2018, while the heavy smoking rate declined continuously from 2008 to 2018. The change trend from 2008 to 2013 is consistent with the previous research results on the prevalence of smoking in China [27]. Moreover, the smoking rate in 2018 (25.33%) in this study is close to the overall smoking rate of China in 2018 estimated by previous scholars [28]. The latter downward trend may be attributable to the establishment of tobacco control laws and regulations, increases in tobacco tax rates and prices, and prohibition of tobacco advertising along with other measures implemented by the Chinese government to curb tobacco use [29]. Additionally, a general increase in health awareness and adoption of healthier living habits as a result of social and economic development may have contributed to the decline. However, there is still no national legislation on smoking in China and the smoking control standards set by the WHO have not been reached. The smoking rate was higher in rural areas than in urban areas in 2008, regardless of whether the smoking was light, moderate, or heavy. However, the trend was reversed in 2018. Similarly, the incidence of “smoking and drinking” in rural areas was higher than that in urban areas in 2008 and 2013, but lower than that in urban areas in 2018. This may be related to the acceleration of urbanization and the influx of rural residents into cities in recent years. At the end of 2018, 59.58% of permanent residents in China lived in urban areas, representing an increase of 8.31% from 2011; meanwhile, 43.37% of registered residents lived in cities, representing an increase of 3.47% from 2015 [30]. Thus, the higher smoking rate in urban as compared to rural areas in 2018 may be the result of a combination of the more frequent social interactions among city dwellers and the large number of smokers from rural areas who migrated to cities.

The rate of alcohol use increased continuously between 2008 and 2018 while the rate of excessive drinking increased from 2008 to 2013 but then declined from 2013 to 2018, consistent with previous reports [31, 32]. Similar to the trend of alcohol consumption, the incidence of “smoking and drinking” also continued to increase between 2008 and 2018. However, in some areas of China, the drinking rate, including excessive drinking rate, continued to decline from 2008 to 2018, which is inconsistent with our research results [33]. In terms of urban-rural trend comparison, although drinking and excessive drinking rates were higher in rural as compared to urban areas from 2008 to 2018, from 2008 to 2013, the rates increased more precipitously in urban areas such that by 2018, the difference between the 2 locations had shrunk. In Chinese culture, drinking is an important way to relieve stress and socialize, and many people regard drinking as a normal habit in rural areas or as serving a social facilitator role in city life. Thus, in order to reduce the rates of alcohol-related diseases and injuries, regulatory policies restricting alcohol use as well as health education are needed. The incidence of “smoking and drinking” increased from 2008 to 2013, and then from 2013 to 2018, regardless of the overall incidence, urban incidence or rural incidence. In previous studies, few scholars calculated the incidence of “both smoking and drinking”, and the analysis of its continuous growth trend found in this study further confirmed the severity of the current situation of smoking and drinking and the necessity of controlling smoking and drinking.

Income level was one of the significant factors influencing the rate and quantity of smoking and drinking. Smoking rate was reported to be higher among people who were poor than among the wealthy [34, 35]. However, in accordance with previous findings [36], we found that income level was positively associated with smoking rate, with the highest income group having more smokers, especially moderate and heavy smokers. The rate of alcohol use—especially excessive drinking—increased with income level, which is supported by earlier studies [15, 37, 38]. People with very high income were also more likely to both smoke and drink than people with low very income. A possible explanation for these observations is the rapid economic development in China and associated increase in income levels, which has led to increased discretionary spending on tobacco and alcohol, especially among people who lack awareness of the adverse health consequences.

We found that education level was also one of the significant influencing factors of smoking and drinking rates: respondents with a higher education level were less likely to smoke and drink (and engage in heavy smoking or excessive drinking) than those with a lower education level, which is consistent with earlier findings [12–14, 39]. A higher educational level may be associated with a greater capacity for translating information into behaviors—in this case, controlling smoking and alcohol consumption. Therefore, improving education level is one strategy to increase health awareness and discourage tobacco and alcohol use in the Chinese population. Employment status was also a significant influencing factor of smoking and drinking behavior: both smoking, drinking,” smoking and drinking” rates were all higher in employed respondents compared to those who were unemployed or retired, which could be related to pressure to socialize and engage with others (colleagues etc) through drinking. In terms of demographic and health-related variables, older people, men, and married people were more likely to smoke and drink alcohol than young people, women, and unmarried people, which is in agreement with other reports [40–42]. Heavy smoking and excessive drinking were also positively correlated with age. This may be because older people in China typically have a low level of education and are thus less likely to be aware of the negative health effects of smoking and drinking, and instead consider these as normal life activities. Additionally, smoking and drinking in women is poorly regarded in Chinese culture; hence, these behaviors are far more common in men. Finally, people who were in poor health (ie, had chronic disease [s]) were less likely to smoke and drink; this is likely because these individuals were incapable of tolerating the effects of tobacco and alcohol.

The results of our analyses indicate that measures are needed to control smoking and alcohol use and promote healthy behaviors in China. Firstly, the government should use public awareness campaigns targeting people with middle or high income levels or low education level, or those living in urban areas. Secondly, the education level of people in rural areas and health awareness among the elderly should be promoted. Thirdly, health facilities (eg, hospitals) should provide more support services such as smoking cessation programs. Fourthly, smoking bans in public places must be strictly enforced by the authorities. Finally, China’s tobacco tax is relatively low compared to that of other countries; increasing taxes on tobacco and alcohol is one way to discourage their consumption.

Our study had certain limitations. Firstly, we analyzed only NHSS data from Jiangsu Province, which may limit the generalizability of the observed trends. Secondly, we examined correlations but were unable to make causal inferences regarding the data. Additionally, our regression analysis focused on individual variables but did not examine the effects of interactions among variables on tobacco and alcohol consumption [43, 44]. Finally, we used logistic regression method (including demographic factors, socio-economic status factors, health factors, etc.) to control the influence of confounding factors. However, although there were many variables, the influence of confounding factors cannot be completely avoided.

## Conclusions

Our results showed that the rate of heavy smoking declined from 2008 to 2018, while overall smoking and light and moderate smoking rates have recently decreased. Similar trends were observed in the rate of excessive alcohol consumption, but overall drinking rate increased continuously from 2008 to 2018. The trends in smoking and drinking rates were similar between urban and rural areas, although smoking rates were initially higher and then lower in rural areas as compared to urban areas. While drinking rates were higher in rural areas throughout the survey period, the rates rose steeply in urban areas such that the difference between the 2 locations had decreased by 2018. The incidence of “smoking and drinking” continued to rise from 2008 to 2018, and this incidence in rural areas was higher than that in urban areas in 2008 and 2013, but lower than that in urban areas in 2018. Socioeconomic factors such as income and education levels and work status as well as demographic and health-related variables influenced smoking and alcohol consumption rates. Our research can provide important evidences for tobacco and alcohol control in China and other similar developing countries. Preventive measures such as increasing education and awareness in key groups and providing technical and support services to control smoking and alcohol use are needed to more effectively promote public health in China.

## Data Availability

Data from this study can be obtained from the corresponding author upon reasonable request.

## Abbreviations

WHO: World Health Organization
NHSS: National Health Service Study
SI: Smoking Index
EQ-5D: European Quality of Life Scale – 5 Dimensions
VAS: Visual Analog Scale.
